# *Angelica dahurica* Extracts Attenuate CFA-Induced Inflammatory Pain via TRPV1 in Mice

**DOI:** 10.1155/2022/4684830

**Published:** 2022-05-23

**Authors:** Chan Zhu, Meiyuan Wang, Jun Guo, Shu Lan Su, Guang Yu, Yan Yang, Yuan Zhou, Zongxiang Tang

**Affiliations:** ^1^School of Medicine, Holistic Integrative Medicine, Nanjing University of Chinese Medicine, Nanjing, Jiangsu Province, China; ^2^Key Laboratory of Chinese Medicine for Prevention and Treatment of Neurological Diseases, Nanjing University of Chinese Medicine, Nanjing, Jiangsu Province, China; ^3^Jiangsu Collaborative Innovation Center of Chinese Medicinal Resources Industrialization, National and Local Collaborative Engineering Center of Chinese Medicinal Resources Industrialization and Formulae Innovative Medicine, Jiangsu Key Laboratory for High Technology Research of TCM Formulae, Nanjing University of Chinese Medicine, Nanjing 210023, Jiangsu Province, China

## Abstract

*Angelica dahurica*, belonging to the family Apiaceae, is a well-known herbal medicine. The roots of *Angelica dahurica* are commonly used for the treatment of headache, toothache, abscess, furunculosis, and acne. However, little is known about their analgesic molecular mechanism underlying pain relief. In this study, we used behavioral tests to assess the analgesic effect of the ADE (*Angelica dahurica* extracts) on CFA (complete Freund's adjuvant)-induced inflammatory pain mice models. TRPV1 (Transient Receptor Potential Cation Channel Subfamily *V* Member 1) protein activity in dorsal root ganglion (DRG) was assessed with a calcium imaging assay. TRPV1 expression was detected with western blot and immunohistochemistry. Then, we examined the constituents of ADE using combined ultra-performance liquid chromatography-quadrupole time-of-light mass spectrometry (UPLC/Q−TOF−MS). Our results showed that ADE effectively attenuated mechanical and thermal hypersensitivities in CFA-induced inflammatory pain model in mice. ADE also significantly reduced the activity and the protein expression of TRPV1 in DRG from CFA mice. Therefore, ADE might be an attractive and suitable analgesic agent for the management of chronic inflammatory pain.

## 1. Background

Pain is normally a transitory unpleasant sensation after a noxious or potentially injurious stimulus, acting as a warning system for tissue protection against injuries and is also a key diagnostic criterion for several acute and chronic medical conditions [1]. Chronic inflammatory pain, one of the most common types of pain, is induced by different chemical mediators released during an inflammatory process, such as bradykinin, prostaglandins, nerve growth factor (NGF), and serotonin (5-HT), which can eventually lead to peripheral and central sensitization. The sensitization of primary nociceptive neurons is a common denominator for all types of inflammatory pain that lead to a state of hyperalgesia and allodynia, typically represented by hypersensitivity to both mechanical and thermal stimuli [2].

Treatment of chronic inflammatory pain still remains a major challenge in clinical practice because of its heterogeneous etiology and the complex underlying pathophysiology mechanisms. Drugs acting on the opioid receptor system or showing nonsteroidal anti-inflammatory mechanisms have been the only successful molecules over the last decades. However, nonsteroidal anti-inflammatory drugs (NSAIDs) are limited by side effects (primarily gastrointestinal and cardiovascular) and insufficient efficacy against the pain of higher intensity. Opioid analgesics may be used for severe inflammatory pain, whereas the risk of side effects, tolerance, and dependence restrict their use. Therefore, there is an urgent need to develop novel and efficient pain medicines that lack side effects to meet the needs of patients.

Traditional Chinese medicine (TCM) has been viewed as a safe treatment and widely used to prevent and cure many diseases under the guidance of the theory of traditional Chinese medical science. *Angelica dahurica* (AD), belonging to the family Apiaceae, is a well-known herbal medicine. The roots of AD (also known as Bai Zhi) are commonly used for the treatment of headache, toothache, abscess, furunculosis, and acne. Pharmacological studies have investigated the effects *Angelicae dahuricae* in terms of anti-inflammatory, analgesic, and antipyretic actions and acute toxicity as a guideline for clinic application [3]. However, little is known about their analgesic molecular mechanism underlying pain relief. In this study, we investigated the antinociceptive activity of *Angelica dahurica* extract (ADE) on cutaneous inflammation pain by CFA-induced mice, and its possible mechanism of the action associated with TRPV1 ion channels was also explored.

## 2. Material and Methods

### 2.1. Preparation of Plant Extract

The roots of *Angelica dahurica* were purchased from Jiangsu Traditional Chinese Medical Hospital (Nanjing, China). The dried roots weighing 200 g were soaked in water (10 times of their total weight) for 1 h and then heated to reflux for 2 h. Eight times of water was added for another 1.5 h refluxing after filtering. The filtered extraction solutions were combined and concentrated using a rotary evaporator at 60°C and then lyophilized. *Angelica dahurica* extracts (ADE) were then prepared into several desired dose concentrations for pharmacological tests.

### 2.2. Analysis of ADE

The chromatographic separation of ADE was performed on ACQUITY UPLC™ liquid chromatographic system (Waters, Milford, MA, USA). An Acquity ^TM^ UPLC BEH C18 column (50 mm × 2.1 mm, i.d., 1.7 *μ*m) was maintained at 30°C with a mobile phase of acetonitrile (A) and 0.1% formic acid aqueous solution (B) using a gradient elution of 0–0.5 min, 5% A; 0.5∼7.5 min, 5% A～30% A; 7.5∼9.0 min, 30% A～40% A; 9.0∼11.0 min, 40% A～50% A; 11.0∼12.0 min, 50% A～80% A; 12.0∼14.0 min, 80% A～80% A; 14.0∼15.0 min, 80% A～70% A; 15.0∼16.0 min, 70% A～60% A; 16.0∼17.0 min, 60% A～50% A; 17.0∼18.0 min, 50% A～35% A; 18.0∼19.0 min, 35% A∼20% A; 19.0∼20.0 min, 20% A～5% A.

Mass spectrometric detection was applied with an electrospray ionization (ESI) ion source. Mass spectra were acquired in the negative-ion mode over a range of m/z 100–1000. The capillary voltage and cone voltage were kept at 3.0 kV and 30 V, respectively. Cone gas flow (50 L/H) and desolvation gas flow (900 L/H) were kept constant throughout the study. The desolvation temperature and ion source temperature were operated at 400°C and 150°C, respectively. Scan time was 0.3 s, and scan interval time was 0.02 s.

### 2.3. Animals

Adult male C57BL/6 mice (aged 6–8 weeks) from the Beijing Vital River Laboratory Animal Technology were used. All experiments involving animals were approved by the Nanjing University of Chinese Medicine Animal Ethics Committee (approval number: ACU170903). The animals were housed in Nanjing University of Chinese Medicine Experimental Animal Center with a temperature-controlled colony room (24 ± 2°C), humidity of 50%–60%, and a 12 h light/12 h dark cycle (lights on 08 : 00 to 20 : 00). Food and water were available ad libitum. All behavioral tests were performed between 09 : 00 and 17 : 00. All experimental procedures were approved by the Animal Care and Use Committee of Nanjing University of Chinese Medicine (Nanjing, China). Mice must adapt to laboratory conditions for at least 2 days prior to testing.

### 2.4. Behavioral Assays

#### 2.4.1. CFA-Induced Chronic Inflammatory Pain

C57BL/6 mice received an intraplantar injection of 20 *μ*l of CFA (Sigma-Aldrich, USA) to the right hind paw and were divided separately into four groups randomly with an equal number (*n* = 10). Four groups of mice were orally administered distilled water, diclofenac sodium (10 mg/kg), or ADE (100 mg/kg, 600 mg/kg) by syringe feeding daily for 14 days after CFA injection, respectively.

#### 2.4.2. Von Frey Mechanical Assay

Mechanical sensitivity in mice was tested using a set of Von Frey filaments (0.008–2 g). Mice were placed on a raised wire mesh grid (6 × 6 mm^2^ apertures) under plastic chambers. The filament was applied to the plantar surface at a vertical angle for up to 2–3 s from the bottom. Fifty percent withdrawal threshold values were determined using the up-down method. Baseline Von Frey measurements were obtained before drug administration or prior to CFA injection, and subsequent measurements were taken at 2, 4, 6, and 8 h and every two days postinjection until the 14^th^ day. Behavioral assessments were conducted during the light cycle at approximately the same time each day. All behavioral analyses were performed blindly.

#### 2.4.3. Radiant Heat Assay

Nociceptive thermal sensitivity was measured by focusing a beam of light on the plantar surface of the hind paw to generate radiant heat. Hind paw withdrawal latency was measured by the method of Hargreaves et al. [4]. Mice were placed in elevated chambers on a plexiglass floor and were allowed to acclimate to the testing room for 30 minutes prior to testing. The radiant heat source (Ugo Basile Plantar Test 37370) was applied to the center of the plantar surface of the hind paw with at least 3 min intervals. The average withdrawal latency of the trials was recorded as the response latency. Baseline latency was determined before drug administration or prior to CFA injection.

#### 2.4.4. Capsaicin-Induced Hyperalgesia

Mice were divided into three groups: control, capsaicin, and capsaicin + osthole. In the control group, 10 *μ*L saline was injected intradermally into the heel pad. Osthole was administered orally 2 hours before capsaicin injection. Capsaicin (10 *μ*L, 500 *μ*M in saline) was injected intradermally into the heel pad with a 0.3 mm diameter needle attached to a Hamilton syringe. The number of flinches and the time of licking the paw were recorded for 25 min.

#### 2.4.5. Tail Immersion Test

For the tail immersion test, the animal was gently restrained, the distal one-third of the tail was immersed into a water bath at 48°C, and the latency to tail flick was recorded.

#### 2.4.6. Cultures of Dissociated DRG Neurons

DRG neurons were isolated using protocols described by our previous experiments [5]. Briefly, The L4–L6 DRGs were collected in cold DH10 (90% DMEM/F-12, 10% FBS, and 1% penicillin-streptomycin-glutamine; Invitrogen) and treated with enzyme solution (5 mg/ml dispase, 1 mg/ml collagenase type I in Hanks' Balanced Salt Solution without Ca^2+^ and Mg^2+^, Invitrogen) at 37°C. Following trituration and centrifugation, cells were resuspended in DH10 and nerve growth factor (50 ng/ml, Upstate), plated on poly-L-lysine (0.5 mg/ml, Stoughton, MA, USA) and laminin (10 *μ*g/ml, Invitrogen) coated glass coverslips, cultured in an incubator (95% O_2_ and 5% CO_2_) at 37°C and used within 48 hours.

#### 2.4.7. Immunostaining of DRG Neurons

Mice were anesthetized with pentobarbital and perfused with 20 ml 0.1 M phosphate buffer solution (PBS; pH 7.4, 4°C) followed by 25 ml fixative (4% formaldehyde in PBS, 4°C). Dorsal root ganglia (DRG) of spine levels L4–L6 were dissected from the perfused mice and then postfixed in fixative at 4°C for 30 min. DRG were cryoprotected in 30% sucrose for more than 24 hour, sectioned with a cryostat at 10 *μ*m, and mounted on slides. Sections were immediately processed for detection of target protein or stored at −20 °C for future use. Sections were incubated in the following solutions: (1) blocking solution (containing 3% fetal bovine serum, 0.1% Triton X-100, and 0.02% sodium azide in PBS) for 1 h at room temperature; (2) primary antibodies (TRPV1 rabbit monoclonal antibody, dilution 1 : 200, Proteintech, 22686-1-AP; neuronal monoclonal antibody, dilution 1 : 500, Abcam, ab104225) in blocking solution at 4°C overnight; (3) PBS, 3 × 10 min each; (4) secondary antibodies (Alexa Fluor 555-labeled Donkey Anti-Rabbit IgG (H + L), 1 : 300, Beyotime, A0453; Alexa Fluor 488-labeled Goat Anti-Rabbit IgG (H + L), 1 : 300, Beyotime, A0423) in blocking solution for 1 h at room temperature; (5) PBS, 3 × 10 min. To calculate the TRPV1-positive cells in DRGs, we set control sections, which were executed under the same conditions and following steps as other sections except that no TRPV1 antibody was added. Then, all the sections were examined with a Carl Zeiss Axio Zoom.V16 fluorescence microscope under the same exposure conditions. The average fluorescence intensity in the control sections was set as the threshold. We calculated the TRPV1 intensity in each cell of different groups by ImageJ. The one whose fluorescence intensity was 1.5 times higher than that of the threshold was considered as a TRPV1-positive cell.

#### 2.4.8. Calcium Imaging

The ipsilateral L4-L6 DRG neurons were loaded with Fura-2-acetoxymethyl ester (Thermo Fisher Scientific) for 25 min in the dark at 37°C in accord with previous studies [6]. After being washed 3 times with PBS, the glass coverslips were placed into a chamber and perfused with a calcium imaging buffer containing 137 mM NaCl, 5.4 mM KCl, 1.2 mM MgCl_2_, 1.2 mM NaH_2_PO_4_, 1 mM CaCl_2_, 10 mM glucose, and 20 mM HEPES (pH 7.4). Ca^2+^ influx was detected by Fura-2 excitation at 340 and 380 nm by a high-speed continuously scanning monochromatic light source (Polychrome V, TILL Photonics, Gräfelfing, Germany). 100 nM capsaicin was added at the indicated time points. Cells were imaged under an Olympus IX57 microscope. All calcium imaging assays were performed by an experimenter blind to the groups.

#### 2.4.9. Western Blot Analysis

Mice were sacrificed by cervical dislocation under isoflurane anesthesia 14 days after the ADE and diclofenac sodium administration. DRG neurons from mice were lysed with RIPA Lysis Buffer (Beyotime, P0013B, China), and protein concentrations were determined using a BCA Protein Assay Kit (Pierce). Proteins (50 *μ*g) were separated in 10% SDS PAGE gel and transferred to PVDF membranes. After 60 minutes of blocking, the membranes were incubated with primary antibody (TRPV1 mouse monoclonal antibody, dilution 1 : 1000, Abcam, ab203103) at 4°C overnight. After washing with TBST, the membrane was incubated with goat anti-mouse antibody (HRP labeled), diluted with 5% nonfat dried milk in TBST, and detected with ECL reagents (Millipore). Blots were scanned with gel and blot imaging system (Biorad, Gel Doc XR+ System), and band densities were compared with Image Lab software. TRPV1 protein levels were normalized against beta-actin, and all experiments were done three times.

#### 2.4.10. Data Analysis

All statistical calculations were performed using the GraphPad Prism 6.0 software. Data are presented as means ± SEMs. Statistical analysis was performed using unpaired *t*-test or one-way analysis of variance (ANOVA) followed by multiple comparisons by post hoc Tukey's test. The designation “*n*” represents the number of animals analyzed. Representative data are from experiments that were replicated biologically at least three times with similar results.

## 3. Results

### 3.1. ADE Attenuated Mechanical and Thermal Hyperalgesia in CFA-Induced Chronic Inflammatory Pain Mice Model

**Figure 1:**
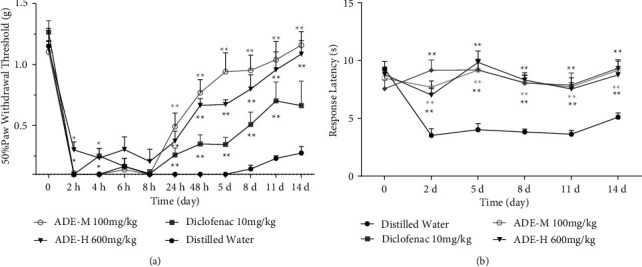
Effects of ADE on the CFA-induced chronic inflammatory pain mice model. Mice were treated with distilled water (control), diclofenac sodium (positive control), ADE-M (100 mg/k), and ADE-H (600 mg/kg) daily after injection of CFA, and then mechanical allodynia (a) and thermal hyperalgesia (b) were tested among the groups. Each point represents the mean ± SEM of the paw withdrawal threshold in response to mechanical or thermal stimuli (^*∗*^*p* < 0.05, ^*∗∗*^*p* < 0.01, ^*∗∗∗*^*p* < 0.001 mean comparison with control group; *n* = 10).

To evaluate the analgesic effect on the chronic pain of ADE, the CFA-induced chronic inflammatory model was adopted. Chronic inflammatory pain is characterized by sensitization of nociceptors resulting in hyperalgesia and allodynia. It can happen with wounds, burns, and infection, evoking a complex series of cellular responses that together are proposed to drive painful hyperalgesic states. Mechanical and thermal hyperalgesia is characterized by increased sensitivity to both heat and mechanical stimulation in the area of injury [7]. In our study, baseline values for the paw thermal and mechanical pain threshold were measured before any treatment. Then, CFA was injected subcutaneously into the plantar surface of the right hind paw for 20 *μ*l per mouse. The control group was treated with distilled water. The positive control group received diclofenac sodium (10 mg/kg) orally. The other two groups received ADE at doses of 100 and 600 mg/kg. As expected, CFA injection induced hypersensitivity to mechanical and heat stimuli in the ipsilateral hind paw during the experimental period (Figures 1(a) and 1(b)). The paw withdrawal threshold in response to mechanical stimuli decreased significantly at 2 h after CFA injection. From 24 h after CFA injection to the last time point assessed (14 days), oral administration of ADE (100 mg/kg and 600 mg/kg) significantly reduced the CFA-induced mechanical hyperalgesia compared to administration of distilled water, the effects of which were superior to oral administration of diclofenac sodium (10 mg/kg). Administration of ADE (100 and 600 mg/kg) and diclofenac sodium also attenuated thermal hyperalgesia in CFA-injected mice as shown in Figure 1(b); mean paw response latency was approximately 3.5 s in the control group which was increased to approximately 8.5 s in the treated groups. This finding indicates that oral administration of ADE can indeed alleviate the mechanical and thermal hyperalgesia in CFA-induced mice model.

### 3.2. ADE Decreased TRPV1 Activity in Isolated DRG Neurons from CFA Mice

**Figure 2:**
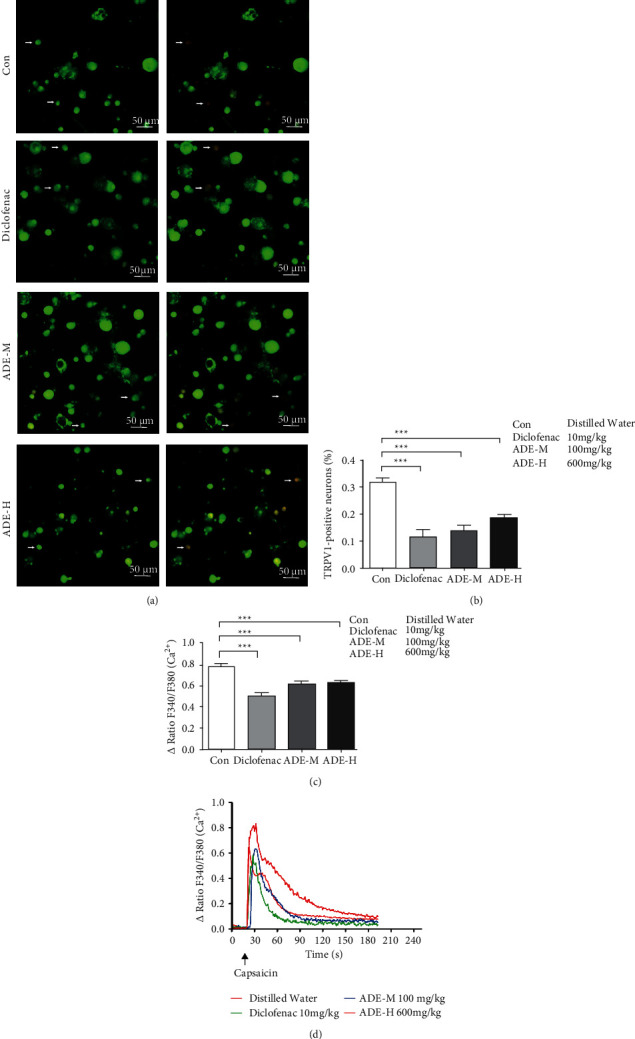
Effect of ADE on the activity of TRPV1. (a) Representative Fura-2 ratio metric images of cultured DRG (L4–L6). Arrow indicates the DRG neurons in response to capsaicin. The color of the neurons switching from green to red indicates an increase in Ca^2+^ influx. (b) Percentage of DRG neurons responding to capsaicin in ipsilateral L4–L6 DRG neurons isolated from different group mice. (c) Activity of ipsilateral L4–L6 DRG neurons responding to capsaicin in neurons isolated from different group mice. (d) Representative traces illustrate that capsaicin elicited Ca^2+^ influx responses in ipsilateral L4–L6 DRG neurons. Each trace corresponds to the change in fluorescence ratio in a single neuron of cultured DRG neurons (^*∗*^*p* < 0.05, ^*∗∗*^*p* < 0.01, ^*∗∗∗*^*p* < 0.001^*∗∗∗*^*p* < 0.001; scale bar: 50 *μ*m; *n* = 3).

To determine how TRPV1 is involved in the analgesic effects of ADE, the activity of TRPV1 in primary cultured lumbar DRG (L4–L6) neurons isolated from CFA mice in four groups was examined. We utilized calcium imaging and detected the intracellular fluorescent calcium level in DRG neurons in response to capsaicin. Representative traces illustrate that capsaicin elicited calcium influx responses in DRG neurons as shown in Figure 2(d). The percentage of capsaicin-responsive neurons in diclofenac sodium, ADE-M, and ADE-H treated mice was significantly reduced by 56.8%, 47.3%, and 48.7%, respectively, as compared to the control group (Figure 2(b)). Additionally, the amplitude of the capsaicin responses DRG neurons from diclofenac sodium, ADE-M, and ADE-H group mice was diminished by 35.6%, 20.8%, and 18.8%, respectively, (Figure 2(c)). These results showed that ADE can decrease TRPV1 activity in isolated sensory neurons from CFA mice.

### 3.3. ADE Decreased TRPV1 Expression in DRG Neurons Isolated from CFA Mice

**Figure 3:**
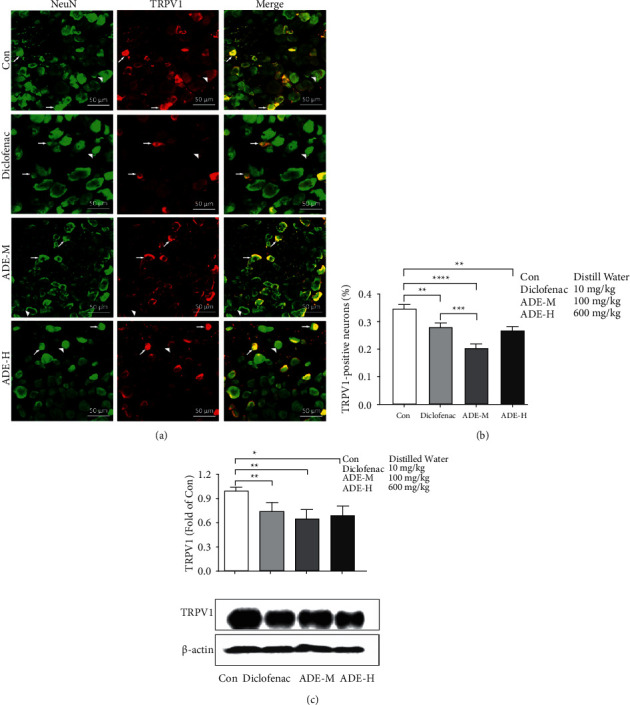
The Effects of ADE on TRPV1 expression in the DRG. (a) Double immunostaining of TRPV1 (red) and NeuN (green) on transverse sections of ipsilateral L4–L6 DRGs from mice of four groups. Arrow and arrowhead indicate neurons with or without TRPV1 expression, respectively. (b) Quantitative data of TRPV1^+^ neurons of four groups were listed. (c) TRPV1 protein levels were analyzed by western blotting (upper panel, summarized bar graph showing band; lower panel, representative western blot bands). Beta-actin was used as internal control for western blot analysis (fold of con refers the fold change of the TRPV1/beta-actin ratio in each group as compared to control group; ^*∗*^*p* < 0.05, ^*∗∗*^*p* < 0.01, ^*∗∗∗*^*p* < 0.001^*∗∗∗*^*p* < 0.001; scale bar: 50 *μ*m; *n* = 3).

Next, to test whether the ADE affects the TRPV1 expression, the TRPV1 expression in primary cultured DRG neurons was examined. CFA mice were orally administered ADE (100, 600 mg/kg), diclofenac sodium (10 mg/kg), or water daily for 14 days. Double immunostaining of TRPV1 and NeuN (a mature neuronal marker) on transverse sections of lumbar DRGs (L4–L6) (Figure 3(a)) shows that the percentage of the TRPV1-positive neurons was significantly decreased in DRGs from CFA mice after administration of diclofenac sodium and ADE (control, 34.7% ± 1.5%; diclofenac sodium, 28 ± 1.3%; ADE-M, 20.5 ± 0.9%; ADE-H, 26.7 ± 1.5% (Figure 3(b)). Western blotting showed that the protein expression of TRPV1 was significantly decreased in the ADE-M and diclofenac sodium groups as compared to the control group (Figure 3(c)).

### 3.4. UPLC/Q-TOF-MS Analysis and Identification of the Constitution of ADE

**Figure 4:**
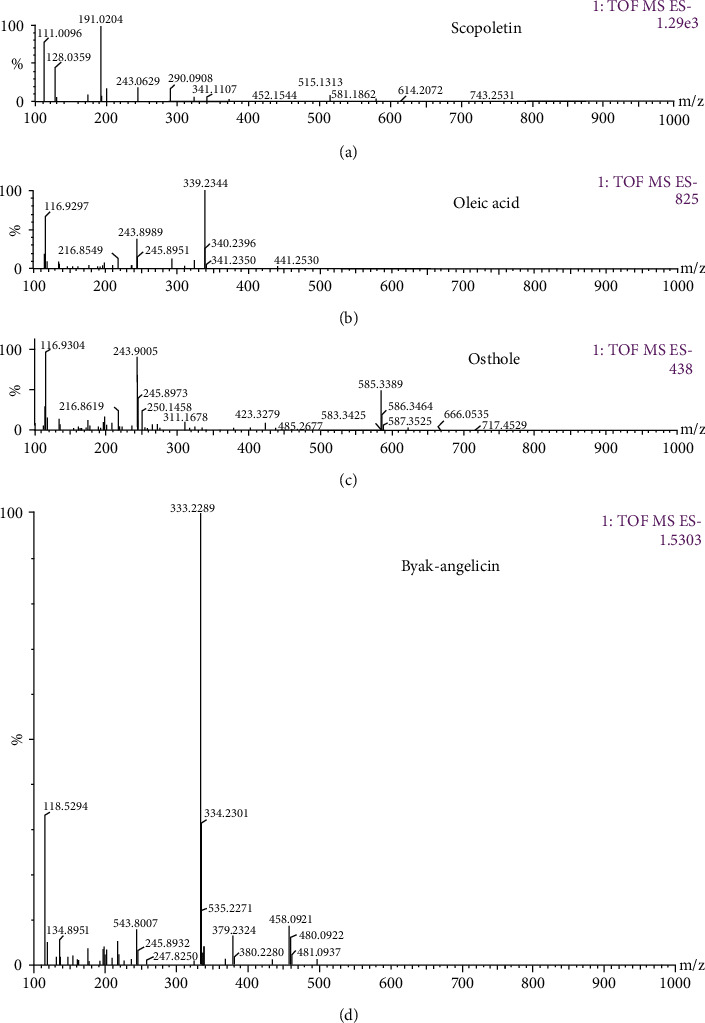
MS spectra of fragment ions of ADE: (a) scopoletin, (b) oleic acid, (c) osthole, and (d) byakangelicin.

Identification of the ADE was based on the retention time, mass assignment, and fragments of corresponding production for the structural identification from the UPLC/Q−TOF−MS analysis platform. In this study, scopoletin, oleic acid, osthole, and byakangelicin were identified by comparing the retention time and mass data with those of the authentic standards. The MS spectra of fragment ions of ADE are shown in Figure 4. The four compounds are summarized in Table 1.

### 3.5. Osthole Attenuated the Noxious Heat and Capsaicin-Induced Pain in Mice

**Figure 5:**
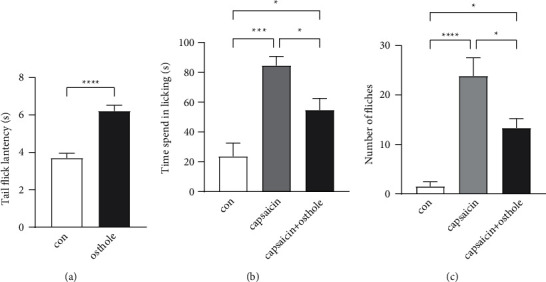
The effect of osthole on noxious heat and capsaicin-induced pain. (a) Comparison of tail-flick latencies after immersion into heated water baths (48°C) following oral administration of osthole. Significantly prolonged tail-flick latency at temperature of 48°C in osthole group as compared to control group. The analysis showed that the time spent in licking (b) and the number of flinches (c) were increased at capsaicin-injected hind paw. Oral administration of osthole can reduce the licking time (b) and flinch number (c) induced by capsaicin-injected (con: control group; 10 *μ*L saline was injected intradermally into the heel pad; ^*∗*^*p* < 0.05, ^*∗∗∗*^*p* < 0.001, ^*∗∗∗∗*^*p* < 0.0001; *n* = 6 per group).

TRPV1 is a nonselective cationic channel activated by painful stimuli such as capsaicin and noxious heat. One of the four identified compounds, osthole, was found to directly inhibit TRPV1 activity in the DRG neurons by our previous study [8]. Here, we performed noxious heat and capsaicin-induced nociceptive behavioral experiments to investigate the role of osthole in the analgesic effect. The latency time for the mice to flick their tails after immersion into heated water baths was recorded in the tail immersion test. As shown in Figure 5(a), the heat stimulus was evaluated at 2 h after mice received oral administration of osthole (20 mg/kg). They exhibited significantly prolonged tail-flick latency at temperature of 48°C as compared to the control group. In addition to thermal sensation, we evaluated the effect of osthole on capsaicin (a TRPV1 agonist) evoked pain. The intraplantar injection of capsaicin produced an intense licking and flinch response toward the injected hind paw. Mice were observed individually for 25 min after capsaicin injection. The time of licking and the number of flinches were reduced significantly after the osthole administration (Figures 5(b) and 5(c)). Therefore, our results show that osthole can reduce noxious heat and capsaicin evoked pain.

## 4. Discussion

There is a growing interest in the utilization of natural products that present fewer side effects for prevention and treatment of pain. *Angelica dahurica* has been traditionally used as a single herb or in combination with other herbs for relieving headache, toothache, supraorbital pain, and rheumatism [9]. To date, the root has been reported to have wound-healing effect [10], antiasthmatic effect [11], antitumor effect [12], and antioxidant activity [13]. Furthermore, recent studies indicated that the extract of *Angelica dahurica* possesses anti-inflammatory and analgesic effect [14]. However, its analgesic molecular mechanism remains unclear.

Our previous study indicated that ADE can potently attenuate heat, capsaicin, and formalin-induced acute pain [15]. To further conﬁrm the analgesia effects of ADE on chronic pain, we established a chronic inﬂammatory pain model by intraplantar injection of CFA in mice. A characteristic symptom of this model of nociception is that it displays hyperalgesia to mechanical and heat stimulation. Our results showed that oral administration of ADE at doses of 100 mg/kg and 600 mg/kg daily could remarkably reduce mechanical and thermal hyperalgesia caused by intraplantar CFA injection. In particular, the antinociceptive effect of ADE treatment on mechanical hyperalgesia was evident when compared to administration of diclofenac sodium (Figures 1(a) and 1(b)), which is a nonsteroidal anti-inflammatory drug used for clinical treatment.

TRPV1 appears to play a critical role in the transduction of noxious chemical and thermal stimuli by sensory nerve endings in peripheral tissues. A significant amount of evidence indicates that TRPV1 is important in the development of inflammatory hyperalgesia. For example, TRPV1 expression in DRG neurons is increased after CFA injection and plays a role in CFA-induced chronic inflammatory pain, such as thermal hyperalgesia and mechanical allodynia [16]. TRPV1 antagonists were known to attenuate two typical symptoms of inflammatory hyperalgesia: thermal and mechanical [17, 18]. Recent evidence supporting the role of TRPV1 in inflammatory pain comes from studies reporting that TRPV1 is essential for analgesia in a mouse model of inflammatory pain [19]. In mouse models, genetic deletion of TRPV1 essentially eliminated thermal hyperalgesia induced by inflammation in two independent TRPV1 knockout lines [20, 21]. Mechanical hyperalgesia was considerably reduced in TRPV1-KO mice in carrageenan-induced hyperalgesia [22]. Considering the close connections of TRPV1 with CFA-induced thermal hyperalgesia and mechanical allodynia, it is reasonable to think that the analgesic effect of ADE may be mediated by TRPV1. In principle, there are two mechanisms by which TRPV1 activity can increase and thus induce thermal hypersensitivity: higher expression levels and increased sensitivity/activity. Indeed, increased expression of TRPV1 at the RNA and protein levels has been reported in inflammatory models [23]. In our present study, calcium imaging assay exhibited that ADE exerted analgesic effects by decreasing TRPV1 activity in isolated DRG neurons from CFA mice (Figure 2). Then, the double-labeling experiments indicate a decrease in the percentage of TRPV1-positive neurons in ADE treated group (Figures 3(a) and 3(b)). Moreover, ADE significantly reduced the protein expression of TRPV1 in DRGs (Figure 3(c)). The results presented here clearly point to an interaction with TRPV1 channels as a possible mechanism of action of ADE. Whether this is direct or indirect interaction, through other intracellular signaling pathways, remains to be elucidated. There is evidence that, regarding disease-related changes in receptor expression, inflammatory mediators such as cytokines, prostaglandin, bradykinin, glutamate, serotonin, and nerve growth factor all have been shown to increase the phosphorylation state of TRPV1, thereby increasing channel activity [24–26]. Specifically, direct phosphorylation of TRPV1 protein by PKC and PKA increases the channel probability and decreases channel desensitization, respectively, and phosphorylation of TRPV1 by the tyrosine kinase Src enhances TRPV1 channel trafficking to the cell surface [27–29].

We also found that the positive control drug diclofenac sodium, which is an NSAID (nonsteroidal anti-inflammatory drug), attenuated the thermal and mechanical hyperalgesia in CFA-induced inﬂammatory pain (Figure 1), reducing the activity and expression of TRPV1 (Figures 2 and 3). NSAIDs' therapeutic benefits are based on their inhibition of cyclooxygenase enzymes and subsequent interference with arachidonic acid pathway metabolites. NSAIDs have also been classified as TRP channel blockers in previous studies [30]. Three regularly used NSAIDs, namely, diclofenac, ketorolac, and xefocam, were found to reduce hyperalgesia generated by TRPV1 agonist capsaicin [31]. Another study found that local NSAID treatment lowered the thermal and mechanical hyperalgesia after TRPA1 or TRPV1 activation [32]. NSAID-serotonin conjugates, such as ibuprofen-5-HT and flurbiprofen-5-HT, have also been shown to inhibit fatty acid amide hydrolase (a membrane-associated intracellular enzyme that degrades endocannabinoids), TRPV1, and COX2, while fenoprofen-5-HT and naproxen-5-HT showed dual inhibitory activity of TRPV1 and COX2 [33]. As a result, our findings are consistent with a previous theory involving NSAIDs' anti-inflammatory and analgesic actions, which result in a reduction in pain-like behavior by inhibiting TRPV1.

Finally, we applied UPLC/Q−TOF−MS to analyze the main analgesia active ingredients in ADE. In the negative-ion mode, scopoletin, oleic acid, osthole, and byakangelicin were identified from ADE. Scopoletin is a reagent with antioxidant, hepatoprotective, and anti-inflammatory activities. Scopoletin was reported to have anti-inflammatory activity in *λ*-carrageenan (Carr)-induced paw edema mouse model of inflammation and can significantly inhibit formalin-induced pain in the late phase [34]. Scopoletin can also ameliorate anxiety-like behaviors in complete Freund's adjuvant-induced mouse model [35]. Oleic acid exhibits an expressive anti-inflammatory effect in croton oil-induced irritant contact dermatitis in mice [36]. A recent study indicates that byakangelicin inhibits IL-1*β*-induced mouse chondrocyte inflammation in vitro and ameliorates murine osteoarthritis in vivo [37]. Osthole was also found to significantly decrease acetic acid, formalin-induced hyperalgesia, and carrageenan-induced paw oedema [38]. In summary, all of the four compounds that we identified from ADE were reported to have anti-inflammatory or analgesic effects, which can partly explain why ADE has a good effect in attenuating chronic inflammation pain. What is more, our previous study indicated that osthole directly inhibited TRPV1 activity in the DRG neurons [8]. Osthole (20 mg/kg) also has been reported to attenuate CCI-induced mechanical and thermal hyperalgesia in mice. Here, we performed nociceptive behavioral experiments to investigate the analgesic effect of osthole. Our results showed that osthole can reduce noxious heat and capsaicin evoked pain.

In conclusion, our data in this study demonstrated for the first time that ADE produced a remarkable inhibition in chronic inflammatory pain models in mice and the possible mechanism of its analgesic action is associated with the inhibition of activities and protein expression of TRPV1. These findings suggest that ADE might be an attractive and suitable therapeutic agent for the management of chronic inflammatory pain.

## Figures and Tables

**Table 1:** The identification results of the constituents of ADE.

No.	Retention time	Name of the compound	Chemical structure	Molecular weight	MS (m/z)
1	0.848	Scopoletin	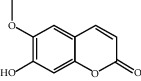	192.168	191.0204 [M-H]-
2	13	Oleic acid	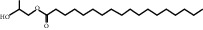	340.5405	339.2344 [M-H]- 116.9304 [M-CH(CH2)7CH3–2H]-
3	14.501	Osthole	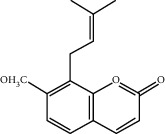	244.29	243.9005 [M-H]-
4	15.217	Byakangelicin	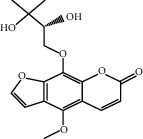	334.31	333.2289 [M-H]- 243.9007 [M-C(CH3)2 OH–OCH3]-

## Data Availability

The datasets used and/or analyzed during the current study are available from the corresponding authors on reasonable request.
